# Video head impulse test in vestibular migraine

**DOI:** 10.1016/j.bjorl.2019.12.009

**Published:** 2020-02-12

**Authors:** Márcio Cavalcante Salmito, Fernando Freitas Ganança

**Affiliations:** Universidade Federal de São Paulo (UNIFESP), São Paulo, SP, Brazil

**Keywords:** Vertigo, Dizziness, Vestibular function tests, Migraine disorders, Head impulse test

## Abstract

**Introduction:**

Vestibular migraine as an entity was described in 1999 and its pathophysiology is still not established. Simultaneously with research to better understand vestibular migraine, there has been an improvement in vestibular function assessment. The video-head impulse test is one of the latest tools to evaluate vestibular function, measuring its vestibular-ocular reflex gain.

**Objective:**

To evaluate vestibular function of vestibular migraine patients using video-head impulse test.

**Methods:**

Cross-sectional case-control study homogeneous by age and gender with vestibular migraine patients according to the 2012–2013 Barany Society/International Headache Society diagnostic criteria submitted to video-head impulse test during intercrisis period.

**Results:**

31 vestibular migraine patients were evaluated with a predominantly female group (90.3%) and mean age of 41 years old. Vestibular function was normal in both patient and control groups. Gain values for horizontal canals were similar between the two groups, but gain values for vertical canals were higher in the group with vestibular migraine (*p* < 0.05). Patients with vestibular migraine felt more dizziness while performing the video-head impulse test than control subjects (*p* < 0.001).

**Conclusions:**

Patients with vestibular migraine present normal vestibular function during intercrisis period when evaluated by video-head impulse test. Vertical canals, however, have higher gains in patients with vestibular migraine than in control subjects. Vestibular migraine patients feel dizziness more often while conducting video-head impulse test.

## Introduction

Dizziness is one of the most common complaints in medical practice, especially in the geriatric age group, with an incidence of up to thirty percent per year,^1^ however, it is possible to reach an etiological diagnosis in most cases.^2^ Vertigo and migraine can coexist in three ways 3, 4, 5: independently, in the same patient; associated with some vertiginous syndrome, such as Meniere's disease; and recurrent vertigo caused by the migraine itself.^5^, ^6^ In the latter case, it is called the Vestibular Migraine (VM). Kayan and Hood found vestibular symptoms more frequent among migrainous patients,^7^ but this VM entity was only recently described in 1999 by Dietrich and Brandt,^8^ and diagnostic criteria have been proposed by Neuhauser in 2001^3^ and recently revised in 2012 jointly by Bárány Society and the International Headache Society (IHS). They are part of the latest International Classification of Headache (ICHD-3), published in 2013 by International Headache Society (HIS).^9^

The pathophysiology of VM is not well established yet. Several mechanisms have been proposed as, for example, the theory of “spreading depression”, alteration of neurotransmitters, and genetic defects in ion channels, these not being mutually exclusive.^4^ The headache due to migraine is classically attributed to a central processing of afferent trigeminal activation in the thalamocortical ascending pathways.^10^, ^11^, ^12^ Ho et al.^13^ expanded the idea that migraine would come only by trigeminal pathways and pain perception to a more complex structure. They theorized the circuit of migraine would include circuitry for processing triggers and premonitory symptoms.^14^, ^15^, ^16^, ^17^ The central vestibular pathways are also part of the circuit of migraine. FMRI studies show that the same regions stimulated by vestibular stimuli are also involved with migraine and pain perception, as posterior insula, orbitofrontal cortex, and anterior and posterior cingulate turns.^18^

Vestibular migraine can occur at any age, with predominance in females.^3^ Patients with VM feature most commonly recurrent attacks of spontaneous vertigo or, sometimes, of positional vertigo. It may occur at the same time or not of a headache.^19^ Associated with vertigo, patients may experience photophobia, phonophobia, visual or other aura.

In parallel with researches to better understand VM, there has been also the improvement of the vestibular function analysis techniques in clinical practice.^20^ Conducting tests to evaluate vestibular function is important to the understanding of the disease and to perform differential diagnoses. Vestibular findings are abnormal only during or shortly after a migraine attack. The presence of alterations in the vestibular tests during the periods between attacks is often indicative of other causes for vertigo.^9^

Currently, the vestibular function is measured indirectly through the physiological vestibular-ocular, vestibular-colic and vestibulospinal reflexes. These reflexes can be tested by electronystagmography and its variants, by cervical and ocular Vestibular Evoked Myogenic Potentials (cVEMPs and oVEMPs) and, more recently, by the Video Head Impulse Test (vHIT).

In the original 1999 article in which vestibular migraine was described, Dieterich and Brandt found vestibular hypofunction in 8.3% of VM patients using caloric test. Other authors, also before the use of the 2012 diagnostic criteria and using nystagmography with caloric testing, found vestibular dysfunction in 10%‒20% of patients with dizziness associated with migrane.^21^, ^22^, ^23^ The results of VEMP in vestibular migraine vary. Some authors found values of latency and wave amplitude equal in VM and control groups^24^, ^25^, ^26^ while others found wave amplitude alterations in patients with VM.^27^, ^28^

After the consensus document of the International Headache Society (IHS) and the Bárány Society, published in 2012, which presented the new diagnostic criteria for VM (Table 1),^9^ it was decided that findings of important changes such as pronounced hearing loss and vestibular dysfunction to caloric test, such as unilateral or bilateral function loss, in the period between attacks, would be indicative of other diseases. These changes should prompt the examiner to consider an alternative diagnosis or the coexistence of two diagnoses.

**Table 1:** Diagnostic criteria for vestibular migraine.^9^

*Vestibular migraine*
A. At least 5 episodes with vestibular symptoms of moderate or severe intensity, lasting 5 min to 72 h
B. Current or previous history of migraine with or without aura according to the International Classification of Headache Disorders (ICHD)
C. One or more migraine features with at least 50% of the vestibular episodes:
Headache with at least two of the following characteristics: one sided location, pulsating quality, moderate or severe pain intensity, aggravation by routine physical activity,
Photophobia and phonophobia,
Visual aura
D. Not better accounted for by another vestibular or ICHD diagnosis
*Probable vestibular migraine*
A. At least 5 episodes with vestibular symptoms of moderate or severe intensity, lasting 5 min to 72 h
B. Only one of the criteria B and C for vestibular migraine is fulfilled (migraine history *or* migraine features during the episode)
C. Not better accounted for by another vestibular or ICHD diagnosis

In 1988, a simple bedside test was described to detect a vestibular hypofunction, characterized by the presence of an eye movement (saccade) after a head tilt (head impulse) done by the examiner while the patient kept his gaze fixed on a target.^29^ This test is known as Head Impulse Test (HIT) or Halmagyi test. HIT has found wide use as a qualitative clinical sign, but it has limitations inherent to any subjective test.^30^ To overcome these limitations, in 2009, a new video oculography system, called Video-Head Impulse Test (vHIT), was developed. It measures the movement of the eye and the head during the impulses and compares them.^30^ The camera is mounted on a very light goggle specially designed to remain firmly adhered to the head. The system is easy to use in a clinical setting, provides an objective measure of the Vestibulo-Ocular Reflex (VOR), and detects covert and overt corrective saccades in patients with abnormal vestibular function. The complete sessions are rapid (approximately 10 min), non-invasive and automated analysis software provides immediate results.

The way the vestibular function is affected by VM has not been fully clarified. Studies with FMRI indicate that it is a central vestibular entity. Peripheral function, however, to date, has not been completely evaluated, because there was no way to verify the function of the vertical semicircular canals. Caloric testing only evaluates the lateral semicircular canals, and not physiologically. The VEMP expanded the peripheral evaluation, particularly evaluating the otolith function. The vHIT is the first test that involves the objective assessment of all the semi-circular canals almost individually, and does it physiologically, i.e., with high frequency head movements, and no studies were found evaluating vestibular migraine using vHIT. In addition to the most complete vestibular evaluation, this new technique shows promise as an easy method to apply and virtually no adverse effects on the evaluation of vestibular function. The high tolerability of this test makes it even more attractive to supplement, or even, in the future, to replace current uncomfortable techniques such as caloric test, feared by many patients who have some vestibular disease.

The objective of this study was to evaluate the vestibular function of patients with VM using the vHIT.

## Method

This is a cross-sectional case-control study, in which v-HIT was carried out in consecutive patients seen at the Vestibular Migraine Clinic of Otorhinolaryngology and Head and Neck Surgery Department of Universidade Federal de São Paulo, all from general neurotology outpatient clinic at the same institution (VM group). All patients were submitted to the test while asymptomatic (between VM episodes) from February 2014 to June 2015 and fulfilled the following criteria: Presence of diagnostic criteria for vestibular migraine or probable VM^9^ as described in Patients whose head circumference allows adjustment of equipment for the examination.

The exclusion criteria were:

1) Not having signed the Informed Consent (IC).
2) Patients with other neurologic diseases, current or past, detected by clinical examination, audiometry or electronystagmography.
3) Conditions that impair the ability to respond to verbal commands.
4) Use of any vestibular sedative medication, such as meclizine, flunarizine or benzodiazepines.

There were also selected individuals to form a control group consisting of healthy individuals with no history of episodic or chronic headache, hearing complaints and/or dizziness, without the use of medicaments, with regular physical ENT examination. It was a convenience sampling (companions of patients, the institution employee and relatives of the researchers or staff). The control group was matched with the patients in the VM group by gender and age.

Individuals in both groups were all subjected to the v-HIT by a single researcher, an ENT physician with training in neurotology with experience to carry out this examination. All tests were performed in Universidade Federal de São Paulo Otorhinolaryngology and Head and Neck Surgery Department with the ICS-impulse® vHIT equipment by Otometrics® company.

To carry out the vHIT, subjects remained seated in a chair, instructed to keep their eyes fixed on one target fixed 120 cm in front, wearing vHIT goggle, which contains an eye movement sensor and another sensor to detect head movements. Head impulses were performed by the examiner on head movement axes that stimulate each of the six semi-circular canals, a total of 20 impulses for each canal. Sensors detect eye and head movements and transcribe them on a graph, visible on the computer screen. The graphics are measured and compared by the computerized equipment program. Gain is calculated by comparing the areas under the head movement and eye movement curves.

The graphics of eye and head movements, when equal, describe the normal situation, classified as gain = 1. When during a head impulse eye movement is less than necessary to keep the gaze fixed on the target, the gain will be less than one, and a second eye movement occurs: corrective saccade. This corrective eye movement takes the gaze back to the target in front of the subject. The equipment considers normal a decrease or increase of up to 0.20 for lateral canals gain and to 0.25 for the vertical canals gain. Saccades were individually identified and counted by the examiner.

If one impulse does not match an appropriate stimulus, the device automatically rejects it. In the present study, were included tests with less than five rejections to obtain 20 accepted stimuli.

The possible vHIT results (outcomes) are: Gain (represented by a number, which may be normal, above or below normal); Presence and number of corrective saccades after head impulses.

The velocity of head impulses was evaluated and compared with the values of obtained gains to identify whether there is a relationship between impulse velocity and VOR gain. Moreover, the average velocity inferred to subjects of both groups was compared, to check the homogeneity of the samples in this regard.

Data were stored and tabulated in Microsoft Excel 2011® program by the main investigator. They were compared between the two groups and statistical calculations were performed: Fisher exact test, Student's *t* test and nonparametric Mann-Whitney. Only the researchers involved in this study had access to the data of each patient.

This study was approved in 2013 by the Ethics Committee of the institution, Platform Brazil – nº 21428313.0.0000.5505.

## Results

The group of patients with VM was composed of 28 women (90.32%) and 3 men (9.68%), aged between 15 and 74 years (mean = 41.58; median = 39). All patients presented in the period between attacks, but one of them did not tolerate the exam and did not have their vHIT data computed in the comparative analysis. The control group consisted of 27 subjects, 24 women (88.89%) and 3 men (11.11%), aged between 17 and 70 years (mean = 41.59; median = 39). The two groups were homogeneous for gender (*p* = 1.000) and age (*p* = 0.998).

**Figure 1:**
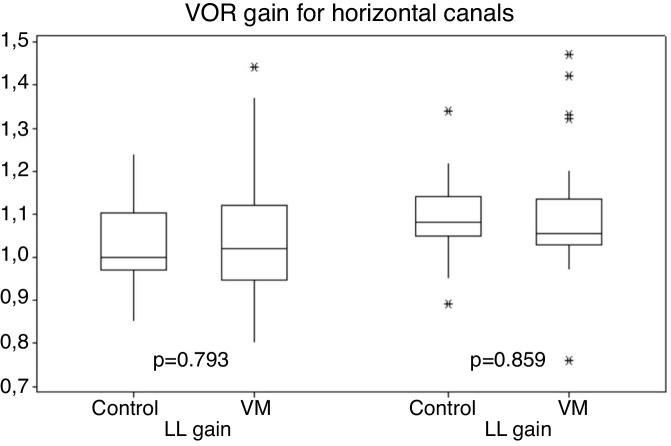
Comparative of horizontal canals gains between VM and control groups. (VM, Vestibular Migraine; LL, Left Lateral canal; RL, Right Lateral canal).

Gains for the horizontal canal tests (right and left canals) ranged from 0.80 to 1.47 for patients with VM, and 0.85–1.34 for individuals in the control group (Fig. 1).

**Figure 2:**
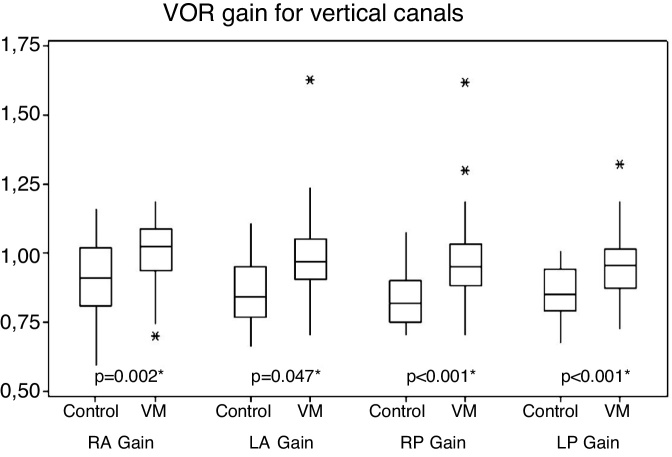
Comparative of vertical canal gains between VM and control groups. (VM, Vestibular Migraine; RA, Right Anterior canal; LA, Left Anterior canal; RP, Right Posterior canal; LP, Left Posterior canal).

For vertical canals, gains ranged from 0.70 to 1.62 in patients with VM and 0.66–1.16 in the control group individuals. There was a statistical difference between the two groups with higher mean values for the VM group (Fig. 2).

There was a negative correlation between the values of head impulses velocity and the obtained gain on all six stimulated canals, with statistical significance in both control and VM groups (Table 2).

**Table 2:** Statistical correlation between performed head impulse velocity and obtained gain in each stimulated canal.

	Correlation	*p*
LL	−40.8%	0.002
RL	−30.5%	0.021
LA	−55.2%	<0.001
RA	−40.4%	0.002
RP	−46.9%	<0.001
LP	−40.7%	0.002

LL, Left Lateral canal; RL, Right Lateral canal; LA, Left Anterior canal; RA, Right Anterior canal; RP, Right Posterior canal; LP, Left Posterior canal.

The mean velocities of head impulses for horizontal canals of VM group were not statistically different from the control group means (Table 3).

**Table 3:** Means of head impulses velocity for horizontal canal tests (º/sec).

Canal tested	n	Mean	*p*
LL control	27	148	0.462
LL VM	30	152	
RL control	27	142	0.859
RL VM	30	152	

LL, Left Lateral canal; RL, Right Lateral canal; VM, Vestibular Migraine.

The mean velocities of head impulses for vertical canals of the VM group were not statistically different from the control group, except for tests of left posterior canal, which had higher averages in the control group (Table 4).

**Table 4:** Mean velocity values of head impulses for vertical canals (°/sec).

Canal tested	n	Mean	*p*
LA control	27	127	0.059
LA VM	30	120	
RA control	27	120	0.122
RA VM	30	115	
RP control	27	124	0.087
RP VM	30	116	
LP control	27	133	0.013*
LP VM	30	121	

LA, Left Anterior canal; RA, Right Anterior canal; RP, Right Posterior canal; LP, Left Posterior canal.

Patients with vestibular migraine felt dizzy while submitted to the vHIT in 22.8% of cases, with statistical difference compared to the control group in which no individual felt dizziness (*p* = 0.004).

The number of saccades occurred after the cephalic stimuli did not differ between groups.

## Discussion

The sample showed a predominance of women and ages vary considerably. Both VM and control groups were statistically homogeneous for these two variables. It is estimated that VM has a higher prevalence in women and can start at any stage of life of people.^31^

Vestibular function measured by the horizontal canal VOR gains was normal in both groups of this study, with no statistical difference between them. The period between attacks of VM is characterized with no symptoms for most patients, and physical examination changes in patients are expected especially during crises, with onset of nystagmus with central characteristics, such as atypical positional nystagmus, spontaneous nystagmus without complaints of dizziness and non-fatigable nystagmus. Vestibular abnormalities during the between crisis periods generally indicate another disease.^9^ The results of normal vestibular function to lateral canals found in this study using vHIT differ from 10% to 20% of vestibular dysfunction found in intercrisis period using the caloric test, which is the traditional way to assess the VOR.^32^ Only a few other studies evaluating vHIT results on VM patients were found. Gain abnormalities were found on VM patients in some studies in 8%‒11% of patients.^33^, ^34^, ^35^

The vertical canals, on the other hand, despite normal mean values ​​of vestibular function measured by vHIT, presented a statistically significant difference between mean VOR gain ​​in patients with VM and those obtained in control subjects, with higher values ​​in VM cases. Possibilities of high gain include the collection errors. Vertical canals are more likely to be affected by artifacts than horizontal canals and strongly depend on horizontal eye position.^36^ Although this knowledge was available after this study, this was probably not the explanation of the difference found because wrongly positioned head can drop, not higher gain values. Possible errors or artifacts that would cause false gain elevation observed by vHIT should, yet, have been distributed similarly between the group of patients with VM and the group of control subjects. Furthermore, it is known that the vHIT leads to slightly higher gains than those obtained by search coil, the gold standard of VOR measurement, due to the fact that an equipment fixed by a rubber band measures head movement. If the equipment is not tightly fixed to the individual's head, there will be an even higher gain. The examiner’s experience could therefore influence the gain value, increasing it when the adjustment of the equipment in the head was done improperly. If the two groups in this study had been examined at different times, a group in the first months of the study and another group in recent months (when the examiner has acquired more experience), the gain values ​​of the group examined before could have an upward bias. Both groups of this study, however, were submitted to vHIT throughout the data collection period.

Another possibility for different gains are different velocities of head impulses. This was observed in this study, where the gain values ​​showed a negative correlation with the impulse velocity, i.e., the higher the impulse velocity, the lower the gain obtained. For horizontal canals, both groups were stimulated with homogeneous velocity of impulses, which makes more consistent the finding of no different gain values ​​of the VM group compared to the control group. For vertical canals, the head impulse velocities were not statistically different in both VM and control groups for the right and left anterior canals and for the right posterior canal, but difference was found in head impulse velocities given to the left posterior canals between the two groups. This difference, however, by low amplitude, seems to have not been the cause of the higher gain values ​​in patients with VM and if it were it would only justify the difference between the right posterior canals of both groups.

Given the possibilities of bias, it becomes challenging to search for an explanation for the VOR gain difference observed between the VM and control groups. One of the pathophysiological mechanisms that have been accepted for migraine is the increased sensitivity of the patient to sensory stimuli. In the case of vestibular system, this increased sensitivity has been demonstrated by studies evaluating the motion detection threshold. Recent studies have shown that VM patients detect less vigorous movements than control subjects, i.e., they demonstrate a lower threshold for detecting movements.^37^, ^38^

A hypersensitive functioning of the vestibular system could result in an exaggerated VOR, suggested by the higher VOR gain found in this work. An abnormally high perception of a head movement, according to Lewis et al.^37^, ^38^ can overestimate the amplitude of the head movement, and thus be the cause of the abnormal motion illusion symptom. The high gain observed by vHIT can be an increased ocular motor response resulting from this perception of a movement greater than the actual. Motion perception however can be affected only in higher processing levels, like time constants or psychophysical experiments.

Another possibility to an increased RVO gain in patients with VM would be a normal vestibular sensitivity, but with elevated responses due to central changes of migraine in the vestibular efferent pathways. However, no studies were found that could scientifically support this hypothesis.

Why was there no gain increase for the horizontal canals? One possible explanation is a neurological mechanism called velocity storage. This phenomenon allows the VOR, after triggered by a stimulus, to continue even in the temporary absence of the same stimulus that triggered it, making it more effective.^39^ A rotating stimulation of the head makes the endolymph circulate in the semicircular canals. If a new rotating stimulation of the head occurs before the endolymph stops circulating, another endolymphatic movement will occur. This movement will be different from the endolymphatic movement due to equal head stimulation, if started from rest. The part of the central nervous system that “understands” this difference to make more accurate vestibular information appears to be that velocity storage.^40^ Velocity storage is a possible mechanism of the head-shaking nystagmus. It has been shown that it is much stronger for the stimulation of horizontal canals than for vertical canals stimulation.^41^ If higher motion detection plays any role in VM vestibular symptoms, the lower expression of this regulatory mechanism (velocity storage) in vertical canals could be an explanation for the higher VOR gain observed only in those canals.

Another difference between horizontal and vertical canals stimulation is that vHIT in the lateral plane is close to a pure rotation, whereas in the vertical planes a linear component is nearly unavoidable so you get some otolith input which could interfere in gain. This component could have been higher in the VM patients as motion sensitive patients tend to stiffen up more their neck, which changes the trajectory of the head in space, which is difficult to control for. If an eye movement is due an extra otolith input or not however makes no difference in achieving the objective of keeping the gaze on target. This should not affect gain values, since linear movements of the eye are detected by vHIT equipment. Only torsional movements are not detected.

The higher prevalence of dizziness complaints during the vHIT observed in the VM group of this study is possibly another indication of this phenomenon of higher sensitivity for the sensory system of motion detection. The rotational stimuli given to the subjects of this study were insufficient to trigger discomfort in individuals of the control group, whereas 25.8% of patients with VM felt some degree of dizziness during the examination, and one of them had the test suspended because she felt too much dizziness. For more stimulating tests such as the caloric test, it is known that patients with VM show more often dizziness (up to four times more) than patients with other neurotological diseases.^42^ It is suggested, therefore, that patients with VM perceive stimuli at a greater intensity sufficient to cause discomfort.

Corrective saccades were observed in both groups of this study, but with no statistically significant difference regarding their frequency. Corrective saccades with normal VOR gain should not occur because a normal VOR must fix our gaze on the target. Despite that, saccades have been seen even in the control group of this study. Minor corrections of gaze could have been due to the target size (about three centimeters) which makes possible small eye movements without leaving the target limits. Another explanation is the presence of artifacts, such as incomplete blink of the eyelid (mini-blinks), which causes the device to identify non-existent eye movements.

## Conclusions

VM patients have normal vestibular function in the period between attacks when evaluated by vHIT. The vertical canals, however, have higher gains in patients with VM than in individuals from a control group. VM patients feel dizziness more often while conducting vHIT.

## Conflicts of interest

The authors declare no conflicts of interest.
